# Characterization of ovine hepatic gene expression profiles in response to *Escherichia coli *lipopolysaccharide using a bovine cDNA microarray

**DOI:** 10.1186/1746-6148-2-34

**Published:** 2006-11-29

**Authors:** Honghe Cao, Leah C Kabaroff, Qiumei You, Alexander Rodriguez, Herman J Boermans, Niel A Karrow

**Affiliations:** 1 Department of Animal and Poultry Science, University of Guelph, Guelph, Ontario, N1G 2W1, Canada; 2 Department of Clinical Studies, University of Guelph, Guelph, Ontario, N1G 2W1, Canada; 3 Department of Biomedical Science, University of Guelph, Guelph, Ontario, N1G 2W1, Canada

## Abstract

**Background:**

During systemic gram-negative bacterial infections, lipopolysaccharide (LPS) ligation to the hepatic Toll-like receptor-4 complex induces the production of hepatic acute phase proteins that are involved in the host response to infection and limit the associated inflammatory process. Identifying the genes that regulate this hepatic response to LPS in ruminants may provide insight into the pathogenesis of bacterial diseases and eventually facilitate breeding of more disease resistant animals. The objective of this research was to profile the expression of ovine hepatic genes in response to *Escherichia coli *LPS challenge (0, 200, 400 ng/kg) using a bovine cDNA microarray and quantitative real-time PCR (qRT-PCR).

**Results:**

Twelve yearling ewes were challenged *iv *with *E. coli *LPS (0, 200, 400 ng/kg) and liver biopsies were collected 4–5 hours post-challenge to assess hepatic gene expression profiles by bovine cDNA microarray and qRT-PCR analyses. The expression of *CD14*, *C3*, *IL12R, NRAMP1*, *SOD *and *IGFBP3 *genes was down regulated, whereas the expression of *ACTHR*, *IFNαR*, *CD1*, *MCP-1 *and *GH *was increased during LPS challenge. With the exception of C3, qRT-PCR analysis of 7 of these genes confirmed the microarray results and demonstrated that GAPDH is not a suitable housekeeping gene in LPS challenged sheep.

**Conclusion:**

We have identified several potentially important genes by bovine cDNA microarray and qRT-PCR analyses that are differentially expressed during the ovine hepatic response to systemic LPS challenge. Their potential role in regulating the inflammatory response to LPS warrants further investigation.

## Background

The innate immune response to gram-negative bacterial infections is initiated by the recognition of lipopolysaccharide (LPS), a principal component of the cell membrane that is released during bacteriolysis. During systemic infections, LPS ligation to the hepatic Toll-like receptor-4 complex induces the production of a wide variety of hepatic acute phase proteins that are involved in the host response to infection and limit the associated inflammatory process [1]. The secretion of pro-inflammatory cytokines for example, plays an important role in the induction of the febrile and hypothalamic-pituitary-adrenal axis responses to LPS [2,3]. The liver's role in LPS removal and metabolism is also well recognized [4], and likely helps to protect the lungs from acute injury during endotoxemia [5]. Given this, the identification of genes that regulate the hepatic response to LPS in ruminants may provide insight into the pathogenesis of bacterial diseases and eventually facilitate breeding of more disease resistant animals.

A number of studies have previously used microarrays to study hepatic gene expression in rats, mice and dogs challenged with LPS; homologous arrays were used in these studies [6-9]. To date however, only two ruminant microarray studies have been performed with bovine cells stimulated with LPS, and these studies were performed *in vitro *[10,11].With respect to sheep, ovine microarrays are not currently available. However, two different groups have constructed bovine immune-related cDNA microarrays that hybridize with ovine cDNA [12,13]. These bovine cDNA microarrays may therefore, be useful for assessing ovine hepatic gene expression in response to systemic LPS challenge.

DNA microarray technology is a powerful and frequently used tool for studying differential gene expression. In comparison to quantitative PCR, one of the significant challenges presented by DNA microarray analysis is having sufficient amounts of high quality RNA that can be labelled and subsequently hybridized onto microarrays. This often requires that animals be euthanized to collect sufficient tissue for RNA extraction, which prohibits the assessment of temporal changes in gene expression *in vivo*. In this study, we amplified total RNA that was isolated from liver biopsy samples and profiled the expression of ovine hepatic genes in response *E. coli *LPS challenge (0, 200, 400 ng/kg) using bovine cDNA microarrays and quantitative real-time PCR (qRT-PCR).

## Results and discussion

### Differentially expressed genes in LPS challenged animals

Gene expression analyses were performed using 8 arrays. Statistical analysis revealed that 11 of genes on the array were differentially expressed between the control and LPS-treated animals (*p *< 0.1) (Table 1). The relative expression of adrenocorticotropic hormone receptor (*ACTHR, p *< 0.07), interferon α receptor (*IFNαR, p *< 0.05), *CD1 *(*p *< 0.03), monocyte-chemoattractant protein 1 (*MCP-1*, *p *< 0.04) and growth hormone (*GH*, *p *< 0.04) genes was increased, while complement component-3 (*C3*, *p *< 0.04), myeloid membrane glycoprotein (*CD14, p *< 0.10), insulin-like growth factor binding protein-3 (*IGFBP3*, *p *< 0.01), interleukin 12 receptor (*IL12R, p *< 0.03), natural resistance-associated macrophage protein-1 (*NRAMP1*, *p *< 0.01) and superoxide dismutase (*SOD*, *p *< 0.08) gene expression was decreased in the LPS-treated animals. Overall, the fold change in gene expression for all of these genes was low (≤ 1.49), even though the signal intensity of *MCP-1, SOD, ACTHR, IL12R *and *NRAMP1 *was relatively high (>5000 pixels) from the microarray slides.

One of the principle complications in microarray analysis of gene expression is the relatively large amount of RNA required for each array. On average, 5–20 μg of total RNA are required per study. This is easily obtained from tissue samples collected from euthanized animals, but is more difficult to obtain from small volume biopsy samples collected from live animals. In this study, the SenseAmp kit (Genisphere Inc. Hatfield, PA) was chosen to amplify total RNA. Goff *et al*. [14] evaluated sense-strand mRNA amplification by quantitative real-time PCR analysis. Their results demonstrated that the SenseAmp kit yields the highest correlation between PCR products before and after amplification, and is also able to accurately amplify partially degraded samples.

### Validated expression of selected genes by quantitative real-time PCR

**Figure 1:**
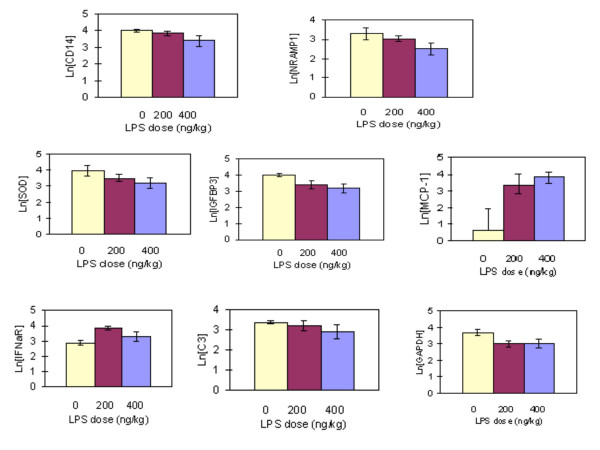
**Real-time PCR validation of the bovine microarray data**. Values are presented as a natural log_e _transformed mean ± SE of 4 sheep per LPS dose group.

Validation of the microarray results by qRT-PCR was performed on the *CD14*, *IFNαR*, *C3*, *NRAMP1, SOD*, *MCP-1*, and *IGFBP3 *genes (Table 2). Two housekeeping genes, *GAPDH *and *RPLPO *were also selected. Results from this analysis generally support the microarray data (Figure 1). Linear orthogonal polynomial contrasts (LOPCS) across dose were significant for *CD14 *(*p *= 0.06), *NRAMP1 *(*p *= 0.05), *SOD *(*p=*0.07)*, IGFBP3 *(*p *= 0.03), and the *GAPDH *housekeeping gene (*p *= 0.05), indicating that the expression of these genes was reduced across LPS doses. *GAPDH *has also recently been reported to not be a reliable hepatic housekeeping gene for rats challenged with LPS [9]. LOPCS across doses was also significant for *MCP-1 *(*p=*0.02), indicating that the expression of this gene was increased across LPS doses. A significant quadratic orthogonal polynomial contrast across dose was also noted for *IFNαR *(*p *= 0.02), indicating that the highest expression of this gene was observed at the 200 ng/kg LPS treatment level. A significant change in the expression of *C3 *and the *RPLPO *housekeeping gene across LPS doses was not observed.

The hepatic genes studied are either involved in LPS recognition, or in regulating the inflammatory response that occurs following LPS recognition. CD14 for example, plays a key role in LPS recognition during bacterial infection. LPS ligation to CD14-TLR4 complex subsequently activates numerous cell types to secrete pro-inflammatory cytokines including IL-6. Recent studies performed with bovine MAC-T cells [15], and rat lung [16] and liver tissues [9] have shown that *CD14 *expression levels were largely unaffected by LPS. An earlier rodent study however, reported up-regulation of *CD14 *3-hours, but not 6-hours post-challenge with 4 μg/kg of LPS administered *i.v *[6]. Previously, our group demonstrated that ovine *CD14 *gene expression increased significantly 2 hours, but not 5 hours after systemic challenge with 200 and 400 ng/kg of LPS [17]. In the present study, *CD14 *gene expression was reduced at the 5-hour sampling time. These results and others suggest that LPS induces tissue-specific and temporal differences in CD14 gene expression.

NRAMP1 functions as a proton/divalent cation transporter in the membranes of the late endosomes/lysosomes, regulating cytoplasmic iron levels in macrophages, and plays a role in host innate immunity [18]. *NRAMP1 *gene expression is dramatically increased in murine macrophages treated with LPS *in vitro *and *in vivo *[19], and its expression is both time- and dose-dependent [20]. A study by Wyllie *et al*. demonstrated using *NRAMP1 *knockout mice that hepatic *NRAMP1 *expression is important for inducing early phase Kupffer cell activation and hepatic inflammation [21]. The LPS dose-dependent down regulation of *NRAMP1 *gene expression observed in the current study may be part of a regulatory mechanism designed to control LPS-induced inflammation in the liver.

As a group of metal-containing enzymes, SODs have a vital anti-oxidant role conferred by their scavenging of the reactive oxygen species [22]. A previous study using rats demonstrated that SOD activity decreased in the liver during the acute phase of an *in vivo *LPS challenge and then increased during the recovery phase. Similar findings were reported with hepatocytes exposed to LPS *in vitro *in the same study [23]. A microarray study using liver tissue from rats challenged with LPS demonstrated induction of the *SOD2 *gene 24 hours post LPS challenge however, no assessment was made at earlier time points [9]. The dose-dependent decrease in *SOD *gene expression that was observed in the present study 4–5 hours post LPS challenge, combined with these previously reported studies, suggest that *SOD *gene expression varies temporally in the liver following LPS challenge.

IGFBPs regulate the bioactivity of mitogenic IGF-I and may also inhibit the growth of certain cell lines by an IGF-I receptor-independent pathway [24]. Priego *et al*. reported that LPS decreased the gene expression of *IGFBP-3 *in the rat liver following *in vivo *challenge [24]. Our results confirm their results using an ovine LPS challenge model.

MCP-1 is an important leukocyte chemoattractant that is involved in recruiting neutrophils and monocytes/macrophages during inflammation. Several studies have shown that LPS induces *MCP-1 *gene expression in various tissues both *in vivo *and *in vitro *[25-27]. Two different LPS studies performed *in vivo *using the rat [9] and canine models [8] made no mention of hepatic MCP-1 induction using microarray analysis, although another chemokine, *MIP-1*, was reportedly induced 4 hours post LPS challenge in the canine model [8]. A recent study however, reported hepatic MCP-1 protein expression in mice challenged with LPS [28]. Our study demonstrates that LPS also induced hepatic MCP-1 gene expression in sheep.

All IFN subtypes are multifunctional cytokines that exhibit differential activities through a common receptor composed of the subunits IFNαR1 and IFNαR2 [29]. In this study we found that the expression of *IFNαR1 *gene was induced after LPS treatment, but the highest expression was observed at the 200 ng/kg LPS treatment level. A study by Severa *et al*., demonstrated that human mature dendritic cells modulate their sensitivity to IFN subtypes by differentially regulating the IFNαR subunits [30]. Future studies on *IFNαR *may help us understand its role during LPS-induced hepatic inflammation.

C3 is a key molecule in both the classical and alternative pathways of the complement cascade. The expression of the *C3 *gene appears to be dependent on LPS dose, sampling time, and cell type. LPS has been reported to induce *C3 *gene expression for example, in a human hepatoma cell line *in vitro *[31] and in human mononuclear phagocytes and human polymorphonuclear leukocytes *in vitro *[32,33]. Others however, have reported that *C3 *gene expression is decreased in monocytes stimulated with LPS [34], and that C3 protein expression follows a bell shaped curve when monocytes are stimulated *in vitro *with LPS between 0.1–100 ng/ml [35]. In the present study we report that *C3 *expression is suppressed in the ovine liver as determined by microarray analysis. Unfortunately, there was insufficient power to validate these results by qRT-PCR analysis.

## Conclusion

In this study, we have identified several potentially important genes that are differentially expressed during the ovine inflammatory response to LPS challenge using bovine cDNA microarray and qRT-PCR analyses. Their potential role in regulating inflammation warrants further investigation. A comparison of these results to those reported in the literatures suggest that hepatic gene expression in response to LPS is dependent on multiple factors including species, tissue, sampling time, the dose and type of LPS.

## Methods

### Liver biopsy trial

Twelve yearling Riduea-Arcott ewe lambs were arbitrary assigned to three groups and challenged with LPS (0, 200 or 400 ng/kg) from *E. coli *serotype 0111:B4 (Sigma Chemical, St. Louis, MO) between 8 and 9 am. Liver biopsies (30–40 mg) were collected between 4 and 5 hours post-challenge, and tissues were immediately placed in RNAlater (Ambion, Austin, TX) and stored at -80°C until total RNA extraction was performed. The doses and biopsy sampling times were based on previous time trial experiment [17].

### RNA extraction and amplification

Total RNA was isolated with Trizol reagent (Invitrogen, Burlington, ON) [17], and amplified using the Genisphere's SenseAmp kit (Genisphere Inc. Hatfield, PA) according to the manufacturer's instructions. Briefly, 0.25 μg of total RNA was used to synthesize first strand cDNA using an oligo (dT) primer and random primer. First strand cDNA was purified then tailed with dTTP using Terminal Deoxynucleotidyl Transferase. The T4 template Oligo was annealed to the 3' end of the cDNA. Klenow enzyme fills in the 3'end of first strand cDNA to produce a double-stranded T7 promoter. Sense-strand RNA (sense RNA) copies of the original starting material were generated during *in vitro *transcription. Amplified sense RNA was quantified using Agilent 2000 Bioanalyzer.

### Construction of a bovine immune-endocrine cDNA microarray

A set of 109 immune, endocrine and inflammation-associated genes was selected for triplicate spotting onto Corning GAPS II slides using a VIRTEK Chip Maker Pro spotter (BioRad, Mississauga, Canada). Positive controls included 5 housekeeping genes (*β-actin, GAPDH, HPRT, PRLPO and β2-microgobulin*), and a serial dilution of pooled bovine genomic DNA. Negative controls included a bacterial gene (VapA) and a partial plasmid sequence of *pACYC177*. All gene products were PCR amplified from either bacterial clones, or liver total RNA. The original clone sets and gene-specific primers were donated by Tao *et al*. [12].

### Microarray hybridization

For each sample, Alexa Fluor 555 or Alexa Fluor 647-labeled cDNAs were generated from 2~2.5 μg of SenseRNA using the SuperScript Indirect cDNA Labeling system (Invitrogen, Burlington, ON). Labelled control animal cDNA was then mixed with labeled cDNA of an animal from either the 200, or 400 ng/kg LPS dose groups, and then hybridized to the array for 18 h in a GeneTAC HybStation (Genomic Solutions, Ann Arbor, MI) using step-down temperatures from 65°C to 50°C in sealed chambers. Following hybridization, the station applies three washes, one with medium stringency buffer, one with high stringency buffer and one with post wash buffer (Genomic Solutions). Slides were finally rinsed briefly at room temperature in 2 × SSC and once in ddH_2_O. Washed microarrays were dried by centrifugation at 1700 rpm for 2 min in a cushioned 50 ml tube. Dye swapping was performed on half of the samples to prevent dye bias. Dried Slides were scanned using GenePix™ 4000 (Axon Instruments Inc. Union City, USA). The images were analyzed and tabulated using GenePix Pro 3.0.

### Microarray data analysis

Microarray data were normalized using LOWESS (Locally Regression and Smoothing Scatterplots) procedure of SAS. The program was obtained from Dr. P. Coussens (Department of Animal Science, Michigan State University). Normalized data were imported into Microsoft Excel, log transformed, and the median blank intensity on a microarray for each dye was subtracted from the respective normalized spot intensity values. These blank corrected values were then used to calculate a mean log expression difference between LPS-treated and control samples. The significance of the values was determined using a two-tailed Student's t-test. The antilog of the mean log expression difference for an individual gene on the array yielded the approximate fold change in expression between cDNA from the LPS-dose groups and control group.

### Quantitative real-time PCR and analysis

To confirm gene expression differences observed from microarray results, qRT-PCR was performed on 9 genes. The primers for housekeeping gene, *GAPDH *and *RPLPO *were developed by Tellam [36]. Other primers were developed using Primer 3 software by our group (Table 2).

qRT-PCR was performed in triplicate for each sample on ABI 7000 Sequence detection system (Applied Biosystems, Streetsville, ON) using default two-step amplification procedures and 2 × SYBR Green Master Mix (Invitrogen, Burlington, ON) in a 25 μl reaction volume according to manufacture instructions. The conditions for the PCR reaction were: 50°C for 2 min, 95°C for 2 min followed by a maximum of 50 cycles of 95°C for 15 sec, annealing temperature for 30 sec and 72°C for 30 sec. The annealing temperatures for genes of interest are included in Table 2. The standard curve method was used to determine relative quantitation of mRNA abundance [37]. Statistical analysis was carried out on the qRT-PCR data using GLM of SAS (SAS 2002, SAS Institute Inc., Gary, NC). Orthogonal polynomial contrasts were performed on the least squares means to identify both linear and quadratic responses across dose. Residual plots were examined to assess homogeneity of variance.

## Authors' contributions

HC carried out the microarray and real-time PCR experiment, participated in construction of the bovine immune-endocrine cDNA microarray and was responsible for manuscript preparation. LCK performed liver biopsy trial and total RNA isolation. QY carried out construction of the bovine immune-endocrine cDNA microarray. AR was responsible for collecting the liver biopsies. HJB was LCK's graduate co-supervisor. NAK facilitated the study, and participated in its design, coordination and analysis, and the liver biopsy sample collection, and helped to draft the manuscript. All authors read and approved the final manuscript.

## Figures and Tables

**Table 1:** Microarray analysis of ovine hepatic gene expression following systemic challenge with 0, 200, or 400 ng/kg LPS

Gene	Description	*p*-value	Expression change
**200/0**			
*CD14*	Myeloid membrane glycoprotein	0.092	-1.49
*ACTHR*	Adrenocortropic hormone receptor	0.063	1.05
*IFNaR*	Interferon receptor α	0.047	1.19
*C3*	Complement component 3	0.038	-1.28
*CD1a.b.d*	CD1	0.026	1.22
*IL12R*	Interleukin 12 receptor	0.021	-1.06
*NRAMP1*	Natural resistance associated macrophage protein	0.002	-1.04
**400/0**			
*C3*	Complement component 3	0.090	-1.16
*SOD*	Superoxide dismutase	0.071	-1.12
*MCP-1*	Monocyte-chemoattractant protein 1	0.035	1.05
*GH*	Growth hormone	0.034	1.10
*IGFBP3*	Insulin-like growth factor binding protein 3	0.007	-1.23

**Table 2:** Primers, and gene accession number, annealing temperature, and product size for quantitative real-time PCR analysis

Gene	Forward & Reverse primer	Accession no	(°C)	(bp)
*GAPDH*	CCTGGAGAAACCTGCCAAGT GCCAAATTCATTGTCGTACCA	TC186924	58	226
*RPLPO*	CAACCCTGAAGTGCTTGACAT AGGCAGATGGATCAGCCA	TC204704	62	220
*CD14*	TGAACATTGCCCAAGCACAC GCCGAGACTGGGATTGTCAG	AY289201	58	101
*C3*	AGAAGCAGAAGCCTGATGGA CCTCGCAGATGTCTTTAGCC	AF038130	60	150
*IFNaR*	CGCATAAGAGCAGAAGAAGGA TGTTCCAGGGGGAGAGATG	U65978	60	150
*NRAMP1*	TTGGCACAGCTATTGCATTC TTCCGCAACCCGTAGTTATC	AF005380	60	121
*SOD*	CGAGGCAAAGGGAGATACAG TCTCCAAACTGATGGACGTG	M81129	60	90
*MCP-1*	GGGTGCTCATTCAGACCATT CATGGGAACTCAAGGAGGAA	L32659	60	126
*IGFBP3*	CAGAGCACAGACACCCAGAA TGCCCGTACTTATCCACACA	AF305199	58	233
